# No evidence for association between polymorphisms in *GRM3 *and schizophrenia

**DOI:** 10.1186/1471-244X-5-23

**Published:** 2005-05-13

**Authors:** Nadine Norton, Hywel J Williams, Sarah Dwyer, Dobril Ivanov, Anna C Preece, Amy Gerrish, Nigel M Williams, Pamela Yerassimou, Stanley Zammit, Michael C O'Donovan, Michael J Owen

**Affiliations:** 1 Department of Psychological Medicine, Wales School of Medicine, Henry Wellcome Building, Cardiff University, Heath Park, Cardiff, UK

## Abstract

**Background:**

Three studies have previously reported data that were interpreted by the authors as supportive of association between schizophrenia and polymorphisms in the gene encoding the metabotropic glutamate receptor *GRM3*.

**Methods:**

In a bid to examine this hypothesis, we examined seven SNPs spanning *GRM3 *in a UK case-control sample (schizophrenic cases n = 674, controls n = 716). These included all SNPs previously reported to be associated, alone or in haplotypes, with schizophrenia in European or European American samples.

**Results:**

Our data showed no evidence for association with single markers, or 2, 3, 4 and 5 marker haplotypes, nor did any specific haplotypes show evidence for association according to previously observed patterns.

**Conclusion:**

Examination of our own data and those of other groups leads us to conclude that at present, *GRM3 *should not be viewed as a gene for which there is replicated evidence for association with schizophrenia.

## Background

Glutamate is the major excitatory neurotransmitter in the central nervous system (CNS) where its effects are mediated through a diverse group of ionotropic receptors [N-methyl D-aspartate receptors (NMDA), alpha-amino-3-hydroxy-5-methyl-4-isoxazole propionate receptors (AMPA), kainate (KA) and delta] and eight metabotropic receptor isoforms. Abnormal glutamatergic transmission has been proposed in schizophrenia, initially from the observation that exposure to NMDA receptor antagonists such as phencyclidine (PCP) can cause symptoms of schizophrenia [1,2] and also relapse in patients with schizophrenia [3]. More recently, agonists of metabotropic glutamate receptors 2 and 3 (GRM2 and GRM3) have been shown to ameliorate the adverse behavioral effects induced by phencyclidine in the mouse [4].

The GRM3 gene (*GRM3*) is therefore an attractive candidate gene for schizophrenia and there have been several reports of allelic association studies examining polymorphisms in this gene [5-8]. These findings, however, do not provide compelling evidence for association. Marti et al., [5] screened the coding exons and flanking intronic sequence of *GRM3 *for polymorphisms in 46 schizophrenics and 46 bipolar patients. Three variants were identified, one of which (+1131C>T, from here on referred to as rs2228595) revealed significant (p = 0.002) evidence for association in a German sample of 265 schizophrenics and 227 controls, a finding that did not replicate in the same study either in a second case-control sample or in a family-based association sample, each if which was also of German origin [5]. The second study of this gene was based upon low density linkage disequilibrium (LD) analysis in a Japanese population [6]. Of six SNPs examined, one, rs1468412, was nominally significantly associated with schizophrenia (p = 0.01). A number of 2 and 3 marker haplotype combinations also yielded support for the association that was surprisingly strong (minimum global p ~0.001) given the sample size was only 100 schizophrenic cases and 100 controls. In the third study [7], rs2228595, which showed un-replicated evidence for increased frequency of the T allele in the earlier German study [5] showed a trend for association of the C allele, (p = 0.08), in a sample of white European American siblings, but not in a second sample of 67 white European American families with one or more affected offspring, or in the two samples combined (616 transmissions observed, 612 expected). Analysis of a further 6 SNPs revealed nominally significant evidence (p = 0.02, uncorrected) for association between an intronic SNP (hcV11245618, from here on referred to as rs6465084) in their first sample. This finding did not replicate in the second Caucasian sample, with any trend being towards excess transmission of the opposite allele, nor in an African American sample of 51 families. The latter sample however yielded evidence for association (p = 0.03) to the allele at rs1468412 that was significantly under-represented in the earlier Japanese study [6]. Haplotype analysis in their Caucasian sib sample revealed a number of related significant 3 and 5 marker haplotypes (minimum global p = 0.006) but no replication data were presented and it is difficult to determine from the presented data to what extent these findings could be expected by chance.

In the most recent study, 7 SNPs were examined in 752 Han Chinese cases and controls [8]. Of the SNPs previously reported to be associated, only rs1468412 was genotyped in this study but this did not reveal evidence for association. However, rs2299225 (which has not been genotyped in any other published datasets) yielded nominally significant evidence (p = 0.03, uncorrected) as did a 3 marker haplotype (global p = 0.0017). Examination of the data suggest that the latter analysis essentially reflects the association at the single locus (frequencies of rs2299225 risk allele = 6.3% in cases, 4.5% in controls; frequency of three marker risk haplotype in cases = 6.2% in cases, 4% in controls), and the extra 'global' significance may have resulted from the use of a statistical test that does not allow for any error in estimating haplotype frequencies.

The hypothesis that this gene may influence schizophrenia susceptibility has gained currency from the observation that homozygosity for one of the alleles of a SNP in *GRM3 *is associated with certain aspects of cognition [7] that have been proposed as intermediate phenotypes for the disorder. Moreover, in that study, homozygosity of the same SNP was also associated *in vivo *with lower indices of synaptic neurotransmission and glutamatatergic function as determined by neuroimaging, and also in post-mortem brain by lower expression of the glial glutamate transporter EAAT2.

Thus the genetic evidence in favour of *GRM3 *is at present ambiguous. What is, however, abundantly clear from two of the studies [6,7] is that it is necessary to invoke genetic heterogeneity (of patterns of LD and/or of the susceptibility alleles *per se*) with respect to ethnicity if the existing data are to be interpreted as supportive of *GRM3 *as a true susceptibility gene for schizophrenia. Therefore in a bid to attempt to provide support for *GRM3 *as a susceptibility gene for schizophrenia, we have focussed on those polymorphisms and haplotypes that have provided significant evidence in Caucasian samples. We typed the SNP showing significance in one of the original German samples [5] as well as the SNPs reported by Egan and colleagues [7], in a large UK based case control sample of 674 cases and 716 controls.

## Methods

### Subjects

All case-control subjects used in this study were unrelated Caucasians born in the UK or Ireland. All cases met DSM-IV criteria for schizophrenia. Consensus diagnoses were made by two raters from all available information following a semi-structured interview, SCAN or PSE [9,10], and examination of case notes. The cases consisted of 456 males and 218 females, average age at collection 44.5 years ± 14.6, whilst the controls consisted of 482 males and 234 females, average age at collection 41.5 years ± 11.5 years. Control individuals were group matched to cases for age, sex, and ethnicity from more than 1400 blood donors recruited from the National Blood Transfusion Service. Individuals on medication are not allowed to donate blood in the UK nor are they remunerated even for expenses. Thus unlike in some countries, donating blood in the UK is entirely an altruistic process that does not tend to enrich for indigents, or people with substance abuses or psychosis. Donors were not screened for the absence of psychiatric illness, as this does not affect the power when a disease has the population prevalence of schizophrenia [11]. Multicentre and Local Research Ethics Committee approval was obtained, and all subjects, both cases and controls, gave written informed consent to participate.

### Genotyping

All SNPs were genotyped using the Sequenom MassARRAY™ system as per the manufacturer's instructions. Assay design and PCR conditions are available on request. All assays were optimised initially by genotyping DNA from 30 CEPH parent-offspring trios from 21 families (Utah residents with ancestry from northern and western Europe), as detailed in the international hapmap project [12]. 46 of these DNA samples were re-genotyped along with the case-control sample to provide a measure of genotyping accuracy. All genotypes were called blind to sample identity and affected status.

### Statistical analysis

All p values are two tailed. Tests of genotypic and allelic association were performed using contingency tables. Haplotype analyses were performed using the EM algorithm and a permutation test as implemented in program EH plus [13]. Linkage disequilibrium values were calculated using the ldmax program within the GOLD software [14].

## Results

Allele frequencies for all SNPs and haplotype frequencies were similar to those reported previously in a Caucasian sample [7]. All markers were in Hardy-Weinberg equilibrium in both cases and controls. Double genotyping of 46 CEPH DNAs for all markers gave 100% concordance (a total of 312 genotypes). Genotype data were available from the Hapmap database for rs917071 and rs1468412. The genotypes we obtained for these individuals were identical to those in the Hapmap database. No significant differences, allelic or genotypic, were observed between cases and controls for any marker even uncorrected for multiple testing (table 1). The only finding approaching nominal significance was for marker rs187993 which yielded a genotypic p-value of 0.06 (2df). No 2, 3, 4 or 5 marker haplotypes yielded evidence for association (data not shown), nor did any of the specific 3 and 5 marker haplotypes reported as significant by Egan et al., [7] under specific 1df tests (table 2).

**Table 1:** *GRM3 *single marker data

		n	**11**	**12**	**22**	**p-value**	**1**	**2**	**p-value**
rs187993	case	590	303	228	59	0.06	834 (0.71)	346 (0.29)	0.11
T/G	control	637	287	288	62		862 (0.68)	412 (0.32)	
rs13242038	case	660	431	213	16	0.19	1075 (0.81)	245 (0.19)	0.60
C/T	control	703	460	214	29		1134 (0.81)	272 (0.19)	
rs917071	case	659	337	272	50	0.61	946 (0.72)	372 (0.28)	0.33
C/T	control	697	373	278	46		1024 (0.73)	370 (0.27)	
rs6465084	case	666	375	246	45	0.93	996 (0.75)	336 (0.25)	0.73
A/G	control	708	406	255	47		1067 (0.75)	349 (0.25)	
rs2228595	case	663	561	98	4	0.85	1220 (0.92)	106 (0.08)	0.64
C/T	control	705	602	100	3		1304 (0.92)	106 (0.08)	
rs1468412	case	663	345	264	54	0.68	954 (0.72)	372 (0.28)	0.41
A/T	control	698	375	274	49		1024 (0.73)	372 (0.27)	
rs7804100	case	652	363	250	39	0.63	976 (0.75)	328 (0.25)	0.46
G/A	control	704	401	269	34		1071 (0.76)	337 (0.24)	

Genotype and allele counts are shown for each SNP. Allele frequencies are shown in brackets. Minor alleles are coded as 2, using coding strand format as per Egan et al., [6]. For comparison between studies, rs2228595 was identified by Marti et al., [8] as +1131C>T and is referred to as snp5 in Egan et al., [6]. rs13242038, rs6465084 and rs7804100 are referred to as hCV2627921, hCV11245618 and hCV2536213 respectively in Egan et al., [6].

**Table 2:** *GRM3 *haplotype data

**SNP**	**Study**	**Haplotype**				
rs187993		2				
rs13242038		1	1			1
rs917071		1	1	1		1
rs6465084			1	1	1	1
rs1468412				1	1	1
rs7804100					1	1

Global P value^a^	Egan et al 2004	0.23	0.05	0.01	0.01	0.01
	Cardiff	0.52	0.80	0.79	0.88	0.82

Best p value ^b^	Egan et al 2004	0.01	0.01	0.01	0.00	0.00
	Cardiff	0.09	0.58	0.60	0.60	0.98

Frequency^c^	Egan et al 2004	0.29	0.57	0.62	0.61	0.53
	Cardiff cases	0.29	0.62	0.66	0.64	0.58
	Cardiff controls	0.32	0.63	0.67	0.65	0.58

Comparison of haplotypes for Cardiff case-control sample and Egan et al., [6] Caucasian sample. ^a ^global p-value, ^b ^best p-value in Egan et al., [6] compared with same haplotype in Cardiff sample, ^c ^Frequency of significant haplotype. In the Cardiff analysis, the no. of controls from 623 to 693 and the no. of cases ranges from 582 to 652.

Allele frequencies and linkage disequilibrium data for the CEPH families were similar to those obtained for the cases and the controls. The LD relationships between markers are given in table 3.

**Table 3:** *GRM3 *linkage disequilibrium data

	rs187993	rs13242038	rs917071	rs6465084	rs2228595	rs1468412	rs7804100
rs187993	x	1	0.94	0.67	0.96	0.57	0.35
rs13242038	0.11	x	0.50	0.38	1	0.32	0.48
rs917071	0.16	0.16	x	0.78	0.91	0.71	0.46
rs6465084	0.07	0.10	0.54	x	0.96	0.86	0.45
rs2228595	0.04	0.18	0.18	0.23	x	0.97	0.60
rs1468412	0.57	0.32	0.50	0.66	0.21	x	0.69
rs7804100	0.35	0.16	0.18	0.20	0.01	0.41	x

LD data for case control sample. D' is shown above the diagonal and r^2 ^is shown below the diagonal.

## Discussion

We sought to replicate specific findings of association between *GRM3 *and schizophrenia in European and European-American samples using a large, UK based case-control sample. However, our data reveal no support for association between any single markers or haplotypes that would represent either replication or indeed novel findings of association. With respect to the specific markers of interest from previous work, marker rs2228595 [5] showed an almost identical allele frequency in cases and controls, with a slight excess (less than 0.5%) of the minor T allele in cases. Thus our data are congruent with the overall combined data reported in the work of Marti and colleagues [5] and also the follow up study of Egan and colleagues [7], and do not support the hypothesis that this allele is associated with schizophrenia. Like Egan and colleagues [7], and unsurprisingly given the postulated ethnic heterogeneity at this locus, we could also not replicate the association between schizophrenia and rs1468412 previously reported in the Japanese sample [6]. However, in contrast to the sibs sample of Egan and colleagues [7], but similar to their family sample, we found a slight excess (0.6%) of the major allele of rs6465084 in controls rather than in cases. Our haplotype analyses were no more supportive of the findings of Egan and colleagues than our single locus analyses. The only specific haplotype reported as associated in that study that approached significance in our sample (nominal p = 0.09, 1df) was the 211 haplotype constructed from rs187993, rs13242038 and rs917071. However, in contrast to the earlier work, we found this to be more common in controls than cases.

There are numerous reasons why in the face of a true association in different samples, different markers and haplotypes might be associated with a disease [15] and therefore it should not in itself be a major concern that the data concerning *GRM3 *follow such a pattern. However, we should nevertheless remain vigilant to the onus in science being rejection of the null hypothesis, and, in the face of confusing patterns of association which may be explicable by heterogeneity, should have explicit criteria for what constitutes replication. A commonsense approach is that exact replication of an allele or haplotype under conventional levels of significance (p < 0.05) can be regarded as replication, with the proviso that if multiple specific hypotheses are tested, this is allowed for. No two studies of *GRM3 *meet this particular criterion for replication. In our own study, we anticipated that rs6465084 would be the most likely marker to replicate in our sample. Although only associated with affected status in one of two Caucasian samples in the study of Egan and colleagues, [7] this marker independently showed evidence for association with measures of glutamate transporter gene expression in post-mortem samples and with neuro-imaging based indices of functionality in controls. This suggested to us that if the findings were not due to chance, they might generalize beyond the specific group of cases reported in that study. Unfortunately, our study provides no support for that prediction.

Alternatively, in the presence of heterogeneity, any study reporting association to novel markers or haplotypes can be considered to provide significant replication provided the analysis allows appropriately (which is not the same as Bonferroni correction) for multiple testing. Given the numbers of markers it is now possible to test in a gene, failure to require this will inevitably lead to accumulation of studies with nominally significant evidence in support of certain markers or haplotypes. It is unclear that any two of the above studies can be considered to provide convincing 'stand alone' evidence for association that would meet this criterion; ours certainly does not. Thus even in the absence of our own data, we do not concur with the suggestion [16] that the evidence for *GRM3 *meets the criteria for significant evidence for association proposed in a meta-analysis of association studies with common variants [17]. However, we do concur with sentiments of Lohmueller et al., [17] whose paper was published "in the light of the seemingly high proportion of false positives in the literature" and who urged the publication of large studies and negative results.

While the current data concerning *GRM3 *do not allow rejection of the null hypothesis, they are insufficient to rule out the possibility of *GRM3 *as a true susceptibility gene for schizophrenia. *GRM3 *spans 221 kb and across this region, the level of linkage disequilibrium across *GRM3 *is moderate (table 3). The current crop of data from the Hapmap includes 63 informative SNPs of which only 28 are redundant using one of the commonly used methods [18]. Thus it does not appear that any of the previous studies, including our own, extract a high proportion of the genetic information relevant to *GRM3*. Whether the existing data suggest that such a study is of sufficient priority to justify embarking upon what is still for most, including ourselves, a costly and time consuming endeavour is clearly a matter for individual researchers. In our view it does not. However, future replication attempts would be greatly facilitated if this challenge were to be taken up by one or more of the groups with data that they themselves consider to be convincing.

## Conclusion

In a bid to attempt to provide support for *GRM3 *as a susceptibility gene for schizophrenia, we have genotyped those polymorphisms that have provided significant evidence in Caucasian samples in a large UK based case control sample. Our data do not support the hypothesis that variation in *GRM3 *is associated with schizophrenia. However, given the moderate level of linkage disequilibrium across this gene, both this study and other current data are insufficient to rule out the possibility that *GRM3 *is a true susceptibility gene for schizophrenia. This will require studies which extract a higher proportion of the genetic data from *GRM3*.

## Competing interests

The author(s) declare that they have no competing interests.

## Authors' contributions

NN, HW, SD, DI, ACP, AG performed laboratory assays. NN performed the data-analysis and drafted the manuscript. NW participated in the design of the study and its coordination. PY performed phenotypic diagnosis. SZ performed sample collection and phenotypic diagnosis. MOD and MJO participated in the design of the study, interpretation of the data, and drafting of the manuscript. All authors read and approved the final manuscript.

**Figure 1:**
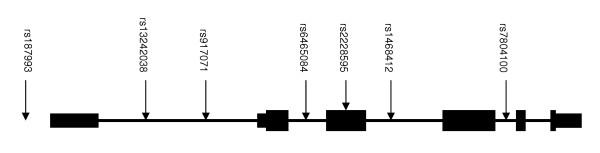
Schematic of *GRM3 *structure with locations of SNPs genotyped in this study. Exons are represented as black boxes. Larger boxing represents coding sequence, and smaller represents UTR. Connecting black lines represent intronic sequence. rs2299225, (significant in Chen et al., [8]) maps to intron 3, but was not typed in this study.

## Pre-publication history

The pre-publication history for this paper can be accessed here:


